# Bioactive Compound Profiling and Antioxidant Activity of *Phytelephas tenuicaulis* and Other Amazonian Fruits

**DOI:** 10.3390/foods13132151

**Published:** 2024-07-07

**Authors:** Elena Coyago-Cruz, David Valenzuela, Aida Guachamin, Gabriela Méndez, Jorge Heredia-Moya, Edwin Vera

**Affiliations:** 1 Carrera de Ingeniería en Biotecnología de los Recursos Naturales, Universidad Politécnica Salesiana, Sede Quito, Campus El Girón, Av. 12 de Octubre N2422 y Wilson, Quito 170143, Ecuador; 2 Maestría en Productos Farmacéuticos Naturales, Universidad Politécnica Salesiana, Quito 170143, Ecuador; 3 Centro de Investigación Biomédica (CENBIO), Facultad de Ciencias de la Salud Eugenio Espejo, Universidad UTE, Quito 170527, Ecuador; 4 Departamento de Ciencias de los Alimentos y Biotecnología, Facultad de Ingeniería Química, Escuela Politécnica Nacional, Av. 12 de Octubre N2422 y Veintimilla, Quito 170524, Ecuador

**Keywords:** bioactive compounds, functional foods, antioxidants, phenolics, carotenoids, organic acid, ascorbic acid

## Abstract

The Amazon region is home to many plant species, many of which have not been studied. The objective was to evaluate the physicochemical properties, bioactive compounds, and antioxidant activity of *Phytelephas tenuicalis* (tintiuk), *Grias neuberthii* (apai), *Euterpe oleracea* (acai), and *Mauritia flexuosa* (brown moriche). Physicochemical analyses were carried out on fresh fruit from local markets. Bioactive compounds (carotenoids, phenolics, vitamin C, and organic acids) were quantified in the freeze-dried pulp by rapid-resolution liquid chromatography (RRLC), and antioxidant activity was determined by ABTS and DPPH assays. The results showed high soluble solids (10.7 °Brix) and ascorbic acid (67.3 mg/100 g DW) in tintiuk; β-carotene (63.4 mg/100 g DW) and malic acid (19.6 g/100 g DW) in brown moriche; quercetin (944.2 mg/100 g DW) and antioxidant activity by ABTS (6.7 mmol ET/100 g DW) in apai; and citric acid (2.1 g/100 g DW) in acai. These results indicate interesting bioactive properties that could increase the consumption of these fruits nationally and internationally, benefiting local farmers and stimulating the development of new products in functional food, medicine, and cosmetics.

## 1. Introduction

Secondary metabolites, which are the products of photosynthesis and whose concentration is influenced by agronomic and environmental characteristics, stand out as the most studied bioactive compounds [1]. Plants are a significant source of bioactive compounds. These include essential and non-essential molecules responsible for modulating various human and plant metabolic processes. When consumed, these compounds have the potential to affect human well-being [2,3,4] positively.

Phenolic compounds and carotenoids are two of the most widely studied bioactive substances. Phenolic compounds, derived from the shikimic acid and/or malonic acid pathways, and carotenoids, non-oxygenated tetraterpenoid hydrocarbons with a structure of forty carbon atoms, play an important role in plant biochemistry. The molecular structure of carotenoids allows them to absorb light in the 400 and 550 nm wavelength range, resulting in a wide range of colours seen in plant tissues, from yellow to red [4,5,6].

Ascorbic acid (vitamin C) and citric acid are primary metabolites and are also part of the bioactive compounds. Vitamin C is involved in metabolic processes in animals and plants, acting as a cofactor for many enzymes. In plants, the main pathway for synthesizing ascorbic acid is the Smirnoff/Wheeler pathway. However, it can also be produced via the *L*-gulose, *D*-galacturonic acid, and myo-inositol pathways. Citric acid, a member of the carboxylic acid group, and other organic acids are major contributors to fruit’s distinctive flavour and aroma [7,8].

Several studies have shown that the regular consumption of fruits and vegetables can play a role in preventing chronic diseases due to the important contribution of the compounds in these plant structures. Vitamin C, for example, acts as a cofactor for important enzymes, has antioxidant properties, and regulates lymphocyte proliferation. Citric acid improves the availability and absorption of minerals and has antioxidant, anti-inflammatory, and anticoagulant properties [7,9,10]. In addition, phenolic compounds protect cells from oxidative stress, promote the production of detoxifying enzymes, and help improve endothelial function, which hinders processes associated with thrombogenesis and lung cancer [3]. Finally, carotenoids offer a range of health benefits, including antioxidant, anti-inflammatory, antidiabetic, anticancer, anti-obesity, neuroprotective, osteoprotective, hepatoprotective, and cardioprotective properties [6,11,12].

On the other hand, due to its unique climatic characteristics, the Amazonian rainforest is home to many plant species, including a wide range of edible fruits used by settlers in the region. For example, *Phytelephas tenuicaulis*, also known as tintiuk or tagua, is a species whose fruits have been used in traditional medicine to treat scorpion stings, snake bites, stingray wounds, abdominal pain, kidney inflammation, and as a diuretic [13,14,15,16,17]. *Grias neuberthii* (apai or python) belongs to the Lecythidaceae family and is a plant endemic to the Amazon region whose seeds, flowers, and pulp are used. This species is known for its antitumour properties [18,19,20]. *Euterpe oleracea* (acai) contains protein, fat, carbohydrates, fibre, minerals, vitamins, phenolic compounds, and carotenoids. It has anti-inflammatory, antioxidant, neuroprotective, anticancer, and antimicrobial activity [21,22,23,24,25,26]. *Mauritia flexuosa* (moriche) is another Amazonian fruit that contains fibre, proteins, carbohydrates, lipids, minerals, vitamins, phenolic compounds, and carotenoids. It has antioxidant, anti-inflammatory, antithrombotic, antiplatelet, anticancer, and antidiabetic activities [20,27,28]. 

The importance of bioactive compounds in promoting human health is widely recognised. However, certain Amazonian fruits have been little studied. In this context, this study was carried out to evaluate the physicochemical characteristics, bioactive compounds (such as carotenoids, phenolic compounds, vitamin C, and organic acids), and antioxidant properties of the edible parts of *Phytelephas tenuicalis*, *Grias neuberthii, Euterpe oleracea,* and *Mauritia flexuosa*. The approach of studying bioactive compounds in Amazonian fruits aims to implement strategies to support their cultivation and explore possible industrial uses while respecting the indigenous communities’ traditional knowledge.

## 2. Materials and Methods

### 2.1. Reagents and Standards

The analytical grade reagents used in this study were Fischer methanol (Fisher Scientific Inc, Madrid, Spain), chloroform, *DL*-homocysteine, *n*-acetyl-*n*,*n*,*n*-trimethylammonium bromide, metaphosphoric acid, ABTS (2,2′-azino-bis-(3-ethylbenzothiazolin-6-sulphonic acid)), potassium persulphate, and DPPH (2,2-diphenyl-1-picrylhydrazyl) which were from Sigma (Merck, Darmstadt, Germany); hydrochloric acid, monobasic potassium phosphate, and sulfuric acid were from Merck (Merck, Darmstadt, Germany); Lobachemie formic acid (Loba Chemie Pvt. Ltd., Mumbai, India); and citric acid BDH (LabTec, Blyth, UK). In addition, HPLC-grade reagents such as Pharmco ethyl acetate and methanol (Greenfield Global, Palo Alto, CA, USA) and Merck acetonitrile (Merck, Darmstadt, Germany) were used. 

Standards such as *L*-(+)-ascorbic acid 99.8%, β-carotene (93.0%), β-cryptoxanthin (97.0%), lycopene (94%), and zeaxanthin were purchased from Sigma-Aldrich (Merck, Darmstadt, Germany), and caffeic acid (98. 0%), gallic acid (100.0%), ferulic acid (100.0%), syringic acid (95.0%), o-coumaric acid (97.0%), *p*-coumaric acid (98. 0%), shikimic acid (99.0%), vanillic acid (97.0%), 3-hydroxybenzoic acid (99.0%), 2,5-dihydroxybenzoic acid (98.0%), quercetin (95.0%), kaemferol (97. 0%), chlorogenic acid (95.0%), naringin (95.0%), citric acid (81.8%), malic acid (99.0%), and *L*-(+)-tartaric acid (99.5%) were purchased from Sigma-Aldrich (Merck, Darmstadt, Germany).

### 2.2. Physicochemical Properties

**Figure 1:**
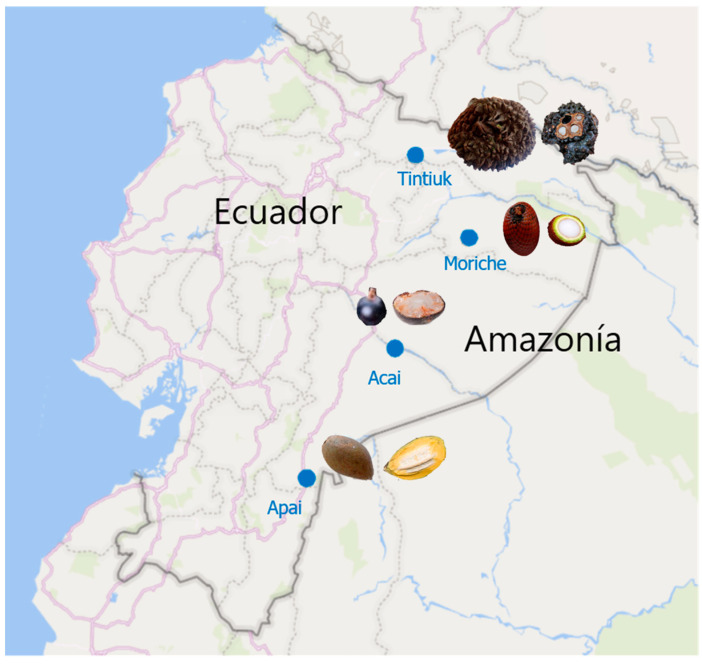
Geographical coordinates of the sampling sites.

The fresh fruits of *Phytelephas tenuicaulis* (tintiuk), *Grias neuberthii* (apai), *Euterpe oleracea* (acai), and *Mauritia flexuosa* (brown moriche) were purchased from local markets in the Amazonian provinces of Ecuador (Figure 1). Thirty fruits were randomly selected, of which ten were used for physicochemical analysis. The remaining edible part of the fruit was homogenised, frozen at −80 °C, and freeze-dried in a Christ Alpha 1–4 LDplus (Martin Gefriertrocknungsanlagen GmbH, Osterode am Harz, Germany). The resulting lyophilised sample was ground to a fine powder using a mortar and pestle and stored in nitrogen-filled amber glass jars until analysed for bioactive compounds and antioxidant activity.

The physicochemical analysis included the analysis of weight and equatorial and lateral diameters of the whole fruit, while in the edible part soluble solids were quantified using a Hitech RHB-32 ATC manual refractometer (G-Won Hitech Co., Ltd., Seoul, Korea), pH using a SevenMulti S47 automatic potentiometer (Mettler Toledo, Columbus, OH, USA), total titratable acid, and moisture in a Memmert Be 20 oven (Memmert GmbH + Co. KG, Barcelona, Spain) at 110 °C to constant weight and the ash in a Thermolyne muffle (Thermo Fisher Scientific, Waltham, MA, USA) at 550 °C to white powder [29]. 

### 2.3. Determination of Bioactive Compounds

#### 2.3.1. Quantification of Carotenoids

Carotenoid extraction was performed in triplicate by mixing 20 mg of the lyophilised powder with 250 µL methanol, 500 µL chloroform, and 250 µL deionised water. The mixture was homogenised in a Vortex Mixer VM-300 (Interbiolab Inc., Orlando, FL, USA), shaken for 2 min in a Fisher Scientific FS60 Ultrasonic (Fisher Scientific Inc., Waltham, MA, USA), and the coloured phase was separated in a MiniSpin microcentrifuge (Eppendorf, Germany) at 1400 rpm for 3 min at 4 °C. This process was repeated until the solid was no longer coloured. The coloured phase obtained was evaporated to dryness on a Buchi TM R-100 rotary evaporator (Fisher Scientific Inc., USA).

The dried extract was redissolved in 40 µL of ethyl acetate. The mixture was centrifuged at 14,000 rpm, 4 °C for 5 min. A total of 30 µL of the supernatant was transferred to a vial insert for analysis on an Agilent 1200 RRLC liquid chromatograph (Agilent Technologies, Mississauga, ON, Canada) coupled to a DAD-UV-VIS detector and an InfinityLab Poroshell 120 EC-C18 column (2.7 µm, 4.6 × 50 mm). The analysis included an injection volume of 20 µL with a mobile phase flow rate of 1 mL/min. A gradient of HPLC grade acetonitrile (A), HPLC grade methanol (B), and HPLC grade ethyl acetate (C) (85% A + 15% B, 0 min; 60% A and 20% B + 20% C, 5 min; 60% A + 20% B + 20% C, 7 min; 85% A + 15% B, 9 min; 85% A + 15% B, 12 min) was used for the analysis. Chromatograms were analysed at 285 nm for phytoene, 250 nm for phytofluene, and 450 nm for α-carotene, β-carotene, β-cryptoxanthin, lycopene, lutein, and violaxanthin using the ChemStation software (version 2.15.26). Identification was performed by comparing the corresponding spectra. Quantification was based on individual calibration curves of 1 mg/mL concentration, injecting volumes of 3, 5, 10, 10, 15, and 20 µL of α-carotene, β-carotene, β-cryptoxanthin, phytoene, lycopene, lutein, and violaxanthin standards, with R2 greater than 0.99 [1]. The concentration of each carotenoid was expressed in milligrams of the carotenoid per 100 g of dry weight (mg/100 g DW).

#### 2.3.2. Quantification of Phenolic Compounds

Phenolic compounds were extracted in triplicate by mixing 40 mg of the lyophilised powder with 1 mL of 80% methanol acidified with 0.1% hydrochloric acid. The mixture was homogenised by vortexing, shaken in an ultrasonic bath for 3 min, centrifuged at 14,000 rpm for 5 min at 4 °C, and the supernatant collected. This process was repeated twice with 500 µL of acidified methanolic solution [30].

The extract obtained was filtered through a 0.45 µm PVDF filter. The resulting filtrate was placed in a vial for analysis in an RRLC coupled to a DAD-UV-VIS detector. A ZORBAX Eclipse Plus C18 column (4.6 mm × 150 mm, 5 µm) conditioned at 30 °C was used for this analysis. The analysis included an injection volume of 20 µL of sample, and a flow rate of 1 mL/min was used, using a gradient of 0.01% formic acid solution (A) and HPLC grade acetonitrile (B). The gradient was as follows: 100% A, 0 min; 95% A + 5% B, 5 min; 50% A + 50% B, 20 min; and a 2 min column clean step. Phenolic identification was performed using the ChemStation software at a wavelength of 280 nm for flavanones and 320 nm for hydroxycinnamic acids and flavones. In addition, the identification included a comparison with each phenolic compound’s corresponding spectra and retention time. Quantification was carried out using a 1 mg/mL concentration calibration curve for which volumes of 3, 5, 10, 15, and 20 µL of the individual standards of caffeic acid, gallic acid, ferulic acid, syringic acid, *o*-coumaric acid, shikimic acid, *p*-coumaric, succinic, vanillic, *p*-hydroxybenzoic, 3-hydroxybenzoic, 2-5, dihydroxybenzoic, quercetin, quercitrin, kaemferol, chrysin, chlorogenic acid, naringin, naringenin, and ethyl gallate were used [1]. The calibration curves showed an R^2^ greater than 0.99. 

#### 2.3.3. Quantification of Vitamin C

The analysis of vitamin C was performed in triplicate. A total of 40 mg of the lyophilised sample was mixed with 200 µL of 0.2% homocysteine and 1.2 mL of 3% metaphosphoric acid. The mixture was vortexed in an ultrasonic bath for 1 min and made up to 2 mL with deionised water. The final mixture was centrifuged at 14,000 rpm for 3 min at 4 °C in a microcentrifuge, and the supernatant was filtered through a 0.45 µm PDVF filter. The filtrate was transferred to a vial for quantification on an RRLC liquid chromatograph coupled to a DAD-UV-VIS detector equipped with a C18 ZORBAX Eclipse XDB 80 AC column (4.6 × 50 mm, 1.8 µm), conditioned at a mobile phase flow rate of 1 mL/min, consisting of 90% 1.5% monobasic potassium phosphate dissolved in water and 10% 1.8% n-acetyl-*n*,*n*,*n*-trimethylammonium bromide dissolved in HPLC grade methanol (solutions were filtered separately). The run time was 20 min, the injection volume 20 µL, and the wavelength 244 nm. 

To construct the calibration curve, 5 mg of ascorbic acid was made up to 5 mL with deionised water, and 3, 5, 10, 10, 15, and 20 µL were injected. The resulting chromatograms were analysed using the ChemStation software [29]. The concentration of vitamin C was expressed in milligrams per 100 g of dry weight (mg/100 g DW).

#### 2.3.4. Quantification of Organic Acid

The extraction of organic acids was performed in triplicate by mixing 40 mg of the lyophilised powder with 1.5 mL of 0.02 N sulphuric acid solution conditioned with 0.05% metaphosphoric acid and 0.02% homocysteine. The mixture was then homogenised by vortexing, shaken in an ultrasonic bath for 3 min, and the supernatant was separated by centrifugation at 14,000 rpm for 5 min at 4 °C. This procedure was repeated. The same procedure was repeated with 500 µL of the acid solution. The collected supernatant was then filtered through a 0.45 µm PVDF filter, and the filtrate was transferred to a vial for analysis on an RRLC liquid chromatograph coupled to a DAD-UV-VIS detector at a wavelength of 210 nm and a YMC-Triart C18 column (150 × 4.6 mm 3 µm, 12 nm). The analysis included an injection volume of 20 µL and a run time of 30 min at a flow rate of 1 mL/min, using a mobile phase of 0.027% sulphuric acid. The chromatograms were analysed at 210 nm using the ChemStation software. Quantification was performed using separately constructed calibration curves based on 5 mg of citric, malic, and tartaric acid, each of which was injected into 5 mL of a 0.02 N sulphuric acid solution in volumes of 3, 5, 10, 15, and 20 µL [30]. The concentrations of each organic acid were expressed in grams of organic acid per 100 g of dry weight (g/100 g DW).

### 2.4. Determination of Antioxidant Activity

#### 2.4.1. Antioxidant Activity by ABTS^•+^ Radical

The extraction for quantifying antioxidant activity by the ABTS^•+^ radical consisted of mixing 20 mg of the lyophilised powder with 400 µL of HPLC-grade methanol and 400 µL of distilled water. The mixture was vortex homogenised and shaken in an ultrasonic bath for 3 min, and the supernatant was separated by microcentrifugation at 14,000 rpm for 5 min at 4 °C. The recovered solid was treated with 5 mL of water. The recovered solid was treated with 560 µL of acetone and 240 µL of distilled water, vortexed, and centrifuged again as described above. The recovered supernatants were mixed and stored under refrigeration until quantification.

For the preparation of the ABTS^•+^ radical, a 1:1 solution of 7 mM ABTS (2,2′-azino-bis-(3-ethylbenzothiazolin-6-sulphonic acid)) was reacted with 2.45 mM potassium persulphate and allowed to stand in the dark for 16 h. The ABTS^•+^ radical solution was then diluted approximately 1:10 with absolute ethanol or until an absorbance of 0.7 at 754 nm was obtained. A 2.5 nM Trolox stock solution with 12.5, 25, 50, and 75% dilutions was used to construct the calibration curve. To quantify the samples, a volume of 10 µL of the final supernatant or standard was added to a 96-well VWR Tissue Culture Plate 96 well-F (Novachen, Pittsburgh, PA, USA) with 200 µL of ABTS^•+^ radical solution. Reading was performed at 270 nm in a spectrophotometer using a Thermo Scientific Multiskan GO microplate reader (Agilent Scientific Instruments, Santa Clara, CA, USA) [30]. Antioxidant activity by the ABTS^•+^ method was expressed in millimoles of trolox equivalent per 100 g dry weight (mmol ET/100 g WD).

#### 2.4.2. Antioxidant Activity by DPPH^•^ Radical

The extraction for quantifying antioxidant activity by DPPH• radical involved mixing 20 mg of the lyophilised powder with 2 mL of HPLC-grade methanol. The mixture was homogenised by vortexing, shaken in an ultrasonic bath for 3 min, and the supernatant was collected by centrifugation at 14,000 rpm for 3 min at 4 °C. 

The calibration curve was prepared by weighing 2 mg of ascorbic acid made up to 2 mL with HPLC grade methanol and diluted to 0, 2, 3, 3, 4, 5, and 6 mg/mL. To form the DPPH^•^ radical, 10 mg of DPPH was weighed and made up to 50 mL with HPLC-grade methanol. For quantification, 20 µL of standard or sample was added to 280 µL of DPPH radical in a 96-well VWR Tissue Culture Plate 96 wells-F (Novachen, USA). In addition, 300 µL of methanol and 300 µL of DPPH^•^ were added as blank in separate wells. The plate was covered with aluminium foil and shaken on a 4310 Shaker Orbital plate shaker (Fisher Scientific, USA) for 30 min. Finally, the plate was read at 560 nm on a BioTek plate spectrophotometer (Scientific Instruments, CA, USA) [30]. Antioxidant activity by the DPPH method was expressed in millimoles of ascorbic acid equivalents per 100 g dry weight (mmol EAA/100 g DW).

### 2.5. Statistical Analysis

Statistical analyses were conducted using Statgraphics Centurion XVII, SigmaPlot (version 14.0), and RStudio (version 4.2.3). The results are expressed as mean ± standard deviation. Mean differences were assessed using Tukey’s test with a significance level set at 0.01 to detect statistically significant variations. Pearson correlations were utilised at a confidence level of 99% to identify potential associations. Furthermore, principal component analysis (PCA) was employed to identify the key variables influencing the outcomes.

## 3. Results

### 3.1. Physicochemical Properties

**Figure 2:**
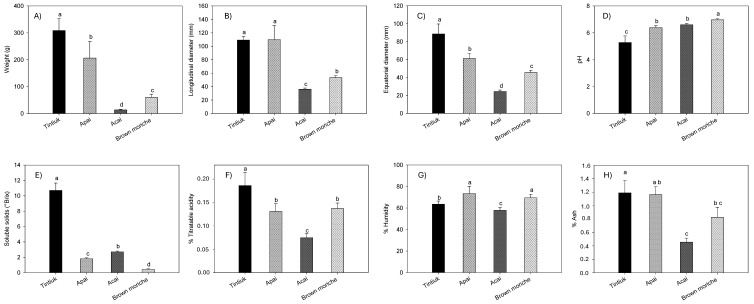
Average values of the weight (**A**), longitudinal diameter (**B**), equatorial diameter (**C**), pH (**D**), soluble solids (**E**), % total titratable acidity (**F**), % moisture (**G**), and % ash (**H**) of the fruits under study. Note: The vertical bars above the columns (average value of n = 20) indicate the standard error, while the different lowercase letters indicate the homogeneous groups according to Tukey’s test with a significance level of *p* < 0.05.

Figure 2 shows the average values of the weight (Figure 2A), longitudinal diameter (Figure 2B), equatorial diameter (Figure 2C), pH (Figure 2D), soluble solids (Figure 2E), % total titratable acidity (Figure 2F), % moisture (Figure 2G), and % ash (Figure 2H) of tintiuk, apai, acai, and brown moriche.

### 3.2. Quantification of Bioactive Compounds

Table 1 shows the average values of the carotenoid and phenolic compound profiles for tintiuk, apai, acai, and brown moriche.

**Figure 3:**
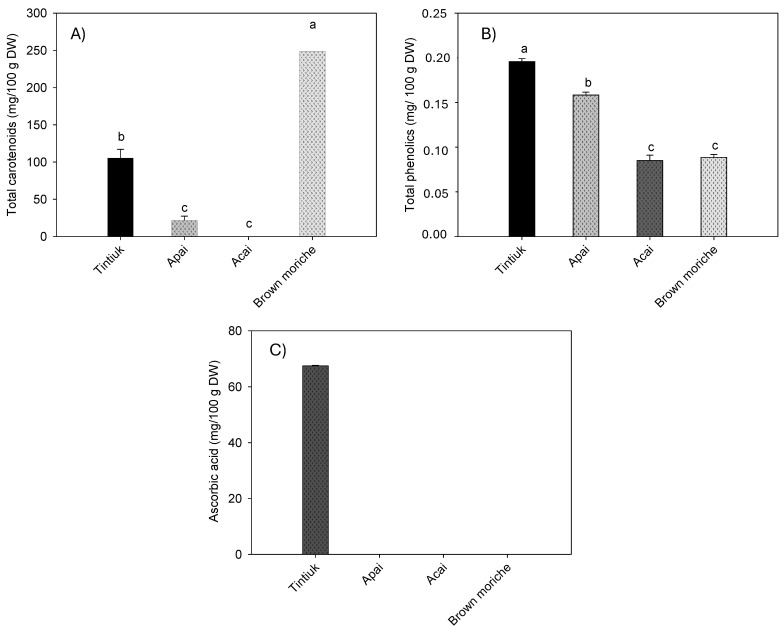
Mean concentrations of the total carotenoids (**A**), total phenolics (**B**), and ascorbic acid (**C**) in the fruits studied. Note: The vertical bars above the columns (mean value of n = 6) indicate the standard error, while the different small letters indicate the homogeneous groups according to Tukey’s test with a significance level of *p* < 0.05.

Figure 3 shows the average concentration of carotenoids (Figure 3A), total phenolics (Figure 3B), and vitamin C (Figure 3C).

**Figure 4:**
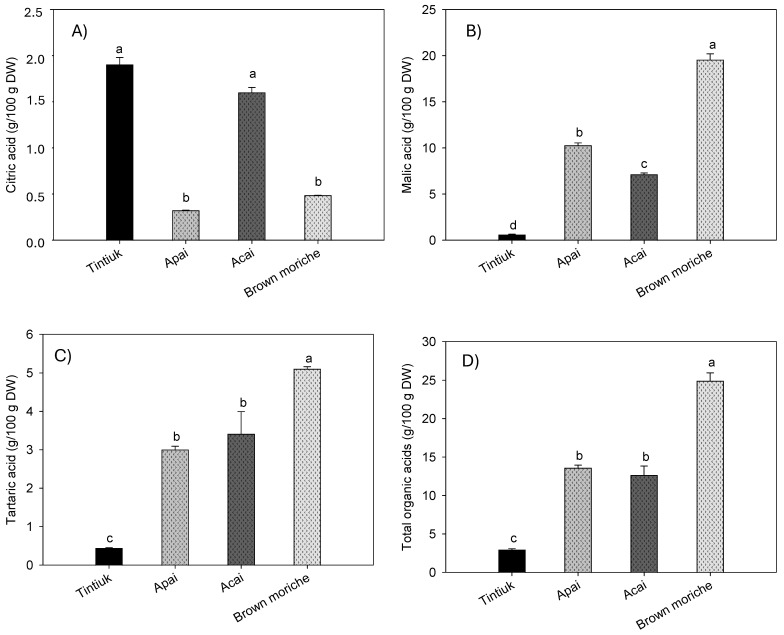
Mean concentrations of the citric acid (**A**), malic acid (**B**), tartaric acid (**C**), and total organic acids (**D**) in the fruits studied. Note: The vertical bars above the columns (mean of n = 6) indicate the standard error, while the different lowercase letters indicate homogeneous groups according to Tukey’s test at a significance level of *p* < 0.05.

Figure 4 shows the average concentrations of citric acid (Figure 4A), malic acid (Figure 4B), tartaric acid (Figure 4C), and total organic acids (Figure 4D) in tintiuk, apai, acai, and brown moriche.

### 3.3. Quantification of Antioxidant Activity

**Figure 5:**
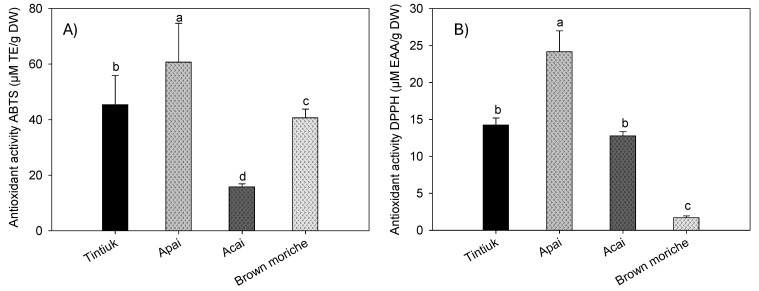
Mean values of the antioxidant activity by ABTS (**A**) and DPPH (**B**) of the fruits studied. Note: The vertical bars above the columns (mean value of n = 9) indicate the standard error, while the different lowercase letters indicate the homogeneous groups according to Tukey’s test with a significance level of *p* < 0.05.

Figure 5 shows the average antioxidant activity of tintiuk, apai, acai, and brown moriche, measured by the ABTS (Figure 5A) and DPPH (Figure 5B) methods.

### 3.4. Statistical Analysis

**Figure 6:**
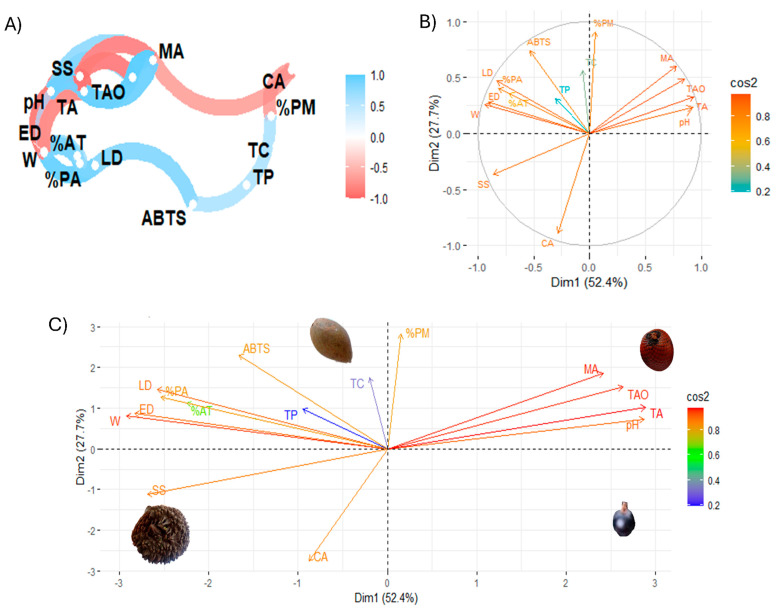
Exploratory multivariate analysis. (**A**) Pearson correlation coefficients; (**B**,**C**) principal component analysis. Note: LD, longitudinal diameter; ED, equatorial diameter; W, weight; SS, soluble solids; %AT, total titratable acid; %PA, % ash; %PM, % moisture; TAO, total organic acids; CA, citric acid; MA, malic acid; TA, tartaric acid; TC, total carotenoid; TP, total phenolic; ABTS, antioxidant activity by ABTS method.

Figure 6 shows Pearson linear correlation coefficients and exploratory multivariate analysis using principal component analysis (PCA).

## 4. Discussion

### 4.1. Physicochemical Properties

The weight values ranged from 14.4 g (acai) to 309.1 g (tintiuk); longitudinal diameter from 36.4 mm (acai) to 110.2 mm (apai); equatorial diameter from 24.5 mm (acai) to 88.8 mm (tintiuk); pH from 5.3 (tintiuk) to 6.9 (brown moriche); soluble solids from 0.5 °Brix (brown moriche) to 10.7 °Brix (tintiuk); titratable acid between 0.08% (acai) and 0.18% (brown moriche); moisture between 58.1% (acai) and 73.6% (apai), and ash between 0.5% (acai) and 1.4% (tintiuk). 

The statistical analysis of pH showed statistical equality between apai and acai, while total titratable acid and moisture showed statistical equality between apai and brown moriche. In addition, the pH values close to neutral suggest that these fruits are more susceptible to microbial attack, as noted by other authors [31]. However, the fruits with higher soluble solid values show higher sugar content, which makes the fruit more attractive to consumers [32].

On the other hand, the fruit weight results in this study were somewhat related to those of other authors, who presented a range between 152 and 784 g for acai [33] and between 40 and 85 g [27,28] and 90 mg GAE/100 g [34] for moriche. However, they differed from the data reported by De-Oliveira and Schwartz (2018), who presented a range between 0.6 and 2.8 g for acai. In terms of size, they were similar to those reported by other authors, who presented a range between 4 and 7 cm equatorial diameter and 7 cm longitudinal diameter for moriche [27,28]. In the case of apai moisture, the values found in this study were in a similar range to those reported by other authors (64.5 to 74.5%) [28], as well as an ash value of 1.0% [35]. In turn, the ash values for moriche were lower than those reported by other authors, who showed a range between 2.1 and 3.0% [25]. The differences and similarities with other studies could be due to agronomic conditions, degree of maturity, environmental conditions, and other factors that contribute to the increase or decrease of the physicochemical parameters of the fruit [1,36]. 

### 4.2. Quantification of Bioactive Compounds

The results showed that brown moriche had high concentrations of β-carotene, acai had high concentrations of caffeic acid, and tintiuk had high concentrations of *p*-hydroxybenzoic acid. In turn, apai showed the highest gallic acid, syringic acid, and quercetin concentrations. In contrast, tintiuk showed the highest concentration of chlorogenic acid, naringenin, and *p*-hydroxybenzoic acid, and acai showed the highest concentration of caffeic acid. 

On the other hand, the acai in this study presented a concentration of lutein and zeinoxanthin, while another study reported the presence of β-carotene, lycopene, astaxanthin, lutein, and zeaxanthin [37]. In turn, the results of individual phenolic compounds presented in this study were consistent with those reported by other authors. For example, in the case of apai, ferulic acid, caffeic acid, synaptic acid, chrysin, and daidzein were reported [33]. Likewise, for acai, the presence of *p*-hydroxybenzoic acid, *p*-coumaric acid, vanillic acid, and ferulic acid, among others, has been reported [25]. The same occurred in moriche, for which the presence of caffeic acid, gallic acid, ferulic acid, luteolin, and kaempferol, among others, was reported [27,28]. These results suggest that species such as tintiuk, which have been little studied, are promising in foods with bioactive potential.

As the sum of the individual carotenoids, the total carotenoids ranged from 1.1 mg/100 g DW (acai) to 66.6 mg/100 g DW (brown moriche). The total phenolics ranged from 395.3 mg/100 g DW (brown moriche) to 1523.6 mg/100 g DW (acai). Vitamin C was found only in tintiuk (67.3 mg/100 g DW), while its presence was not detected in the other fruits.

On the other hand, the results of this study showed higher concentrations of carotenoids than those reported by other authors, who reported a value of 2.2 mg of total carotenoids/100 g in pulp [33] and a value of 295.2 µg of β-carotene/g of fresh pulp in moriche [27]. In turn, differences in ascorbic acid concentration were reported to be 0.01 mg/100 g fresh weight for acai [21] and 25.0 mg/100 g fresh pulp for moriche [27]. However, it is essential to note that the vitamin C concentration of tintiuk was considerable compared to the concentrations of other fruits reported in other studies [29].

The concentration of citric acid ranged from 0.2 g/100 g DW (brown moriche) to 2.1 g/100 g DW (acai). In contrast, the concentration of malic acid ranged from 0.6 g/100 g DW (tintiuk) to 19.6 g/100 g DW (brown moriche). On the other hand, the concentration of tartaric acid ranged from 0.4 g/100 g DW (tintiuk) to 5.1 g/100 g DW (brown moriche). Although no studies were found to compare the results of this study with those of other authors, it can be seen from Figure 4B that malic acid had the highest concentrations in all the fruits analysed. This finding is consistent with that reported by several authors, who point out that this acid is one of the most important organic acids in some fruits and is widely used in the pharmaceutical and cosmetic industries [7,9,10].

### 4.3. Quantification of Antioxidant Activity

The antioxidant activity by the ABTS^•+^ radical ranged from 1.6 mmol ET/100 g DW (acai) to 6.7 mmol ET/100 g DW (apai), and by the DPPH^•^ radical from 0.17 mmol EAA/100 g DW (brown moriche) to 2.4 mmol EAA/g DW (apai). In this sense, the antioxidant activity is the ability of certain substances to prevent or retard the oxidation of molecules by scavenging free radicals and reactive oxygen species. For in vitro determination, it is necessary to isolate or concentrate bioactive compounds with antioxidant properties for subsequent use in methods such as DPPH, FRAP, and ABTS [38,39,40]. This indicates that the species under study have inhibition percentages exceeding 10%, which indicates that even acai and brown moriche, which have low antioxidant activity, have the inhibition of free radicals generated.

The results obtained for acai in this study showed lower values of antioxidant activity by the ABTS^•+^ and DPPH^−^ radicals than those reported by other authors (55.1 µM ET/g fresh weight and 4673.1 EC50 expressed in g pulp/g DPPH^•^, respectively) [23], in turn, 320.3 µM ET/g DW by the DPPH method [37]. For brown moriche, a lower ABTS^•+^ radical antioxidant activity (13.0 µM ET/g DW) [27,41] and a lower DPPH^•^ radical antioxidant activity (1.9 EC50 g sample/g DPPH^•^) and an IC50 of 60% were also found [27,34,41]. 

On the other hand, the difference in antioxidant activity results between the ABTS and DPPH assays can be attributed to the specific reactions measured by each assay. Although both assays assess antioxidant capacity, they do so by different mechanisms. The ABTS assay measures the ability of antioxidants to reduce ABTS radicals, focusing on a single electron transfer reaction. In contrast, the DPPH assay assesses the scavenging of DPPH radicals, which involves a hydrogen atom transfer reaction [42,43]. This distinction leads to variations in results, as certain compounds may react differently with each radical. For example, acai and brown moriche show significant differences between the assays. In addition, the structure/activity relationship in the ABTS assay may not always match that of the DPPH assay, which further contributes to discrepancies in results, as noted by other authors [43].

### 4.4. Statistical Analysis

The multivariate analysis of the study variables, in particular the correlation analysis of the variables (Figure 6A), showed that the total concentration of organic acids is positively correlated with malic acid, while the antioxidant activity by ABTS is negatively correlated with variables such as size and % ash, which could indicate that certain compounds or characteristics present in these samples reduce the antioxidant capacity measured by ABTS. On the other hand, pH and total titratable acidity were correlated, suggesting that pH and acidity may have an important influence on antioxidant activity. In this regard, an inverse relationship has been observed between citric acid and malic acid, which can be explained by the interdependence in the biosynthesis of these organic acids. The formation of malate requires the use of citrate, so an increase in the concentration of one acid leads to a decrease in the concentration of the other [44]. Furthermore, an inverse correlation was found between soluble solids and fruit size, in line with previous research on other fruits [5]. Similarly, soluble solids showed an inverse relationship with organic acids, as shown in other studies [45]. Finally, a direct relationship was shown between total phenolic compounds and total carotenoids, as indicated by other authors [46,47]

Figure 6B is a biplot graph showing the relationship between the variables in the space of the first two principal components (Dim 1 and Dim2), with the intensity of the colour indicating the quality of the representation (cos2). Dim 1 (52.4%) and Dim2 (27.7%) explain a significant percentage of the total variance of the data. This indicates that variables such as the concentration of total organic acids, malic acid, and tartaric acid are highly correlated with Dim 1, suggesting that this principal component mainly captures the variance associated with antioxidant activity and acids. In turn, ABTS and % ash have an opposite orientation in the biplot, indicating that these parameters are inversely related. In addition, variables such as citric acid and soluble solids are closer to the origin, suggesting a lower contribution to the principal component.

Figure 6C is a plot that projects the samples into the space defined by the first two principal components. This figure shows the location in different quadrants of different characteristics. For example, total organic and malic acid are separated from the other variables. Furthermore, this analysis showed that physicochemical parameters and organic acids strongly influence the samples under study, while carotenoids and phenolic compounds have a lower incidence. The biplot principal component analysis showed that tintiuk has a higher incidence of physicochemical characteristics, mainly soluble solids; apai of phenolic compounds, carotenoids, and antioxidant activity; and brown moriche of organic acids. At the same time, acai is not influenced mainly by a specific group.

## 5. Conclusions

Although certain Amazonian fruits are popular locally, only part of their properties have been studied, such as phenolic compounds and their antioxidant activity, but there are still gaps in the research on ascorbic acid and organic acids. Thus, the results of this study showed that tintiuk is a fruit with a high content of soluble solids, ash, and vitamin C); brown moriche, which has a high pH, a high percentage of total titratable acid, β-carotene, malic acid, and tartaric acid; apai, which has a high moisture content, phenolic compounds, and antioxidant activity; and acai has a high concentration of citric acid. These results may encourage future research and promote the consumption and sustainable use of these Amazonian fruits, which can benefit local communities and industry.

## Figures and Tables

**Table 1:** Mean values of carotenoid and phenolic profile.

		Tintiuk			Apai			Acai			Brown Moriche		
Carotenoids (mg/100 g DW)	α-Carotene	4.4 ± 0.5			3.0 ± 0.3								
	β-Carotene	22.4 ± 2.3			6.7 ± 1.8						63.4 ± 3.3		
	β-Cryptoxanthin	7.1 ± 0.0			5.8 ± 0.5								
	Luteoxanthin	4.9 ± 0.8											
	ζ-Carotene	6.6 ± 0.9											
	Lutein				1.4 ± 0.1			1.1 ± 0.1					
	Zeinoxanthin				1.3 ± 0.1			3.2 ± 0.1					
Phenolics (mg/100 g DW)	Gallic acid	12.4 ± 0.2			61.7 ± 2.3			25.8 ± 0.6			6.0 ± 0.3		
	Syringic acid				81.6 ± 1.1			11.2 ± 0.6			12.5 ± 0.5		
	Chlorogenic acid	48.6 ± 2.2			35.5 ± 0.5			15.0 ± 2.6			7.7 ± 0.5		
	Caffeic acid	179.9 ± 4.8			175.8 ± 20.6			397.7 ± 0.5			23.0 ± 0.3		
	Naringenin	28.2 ± 1.6											
	*p*-Hydroxybenzoic acid	398.3 ± 32.5			224.7 ± 13.7			212.8 ± 17.6			346.2 ± 17.0		
	Quercetin				944.2 ± 19.2								

## Data Availability

The original contributions presented in the study are included in the article, further inquiries can be directed to the corresponding author.
